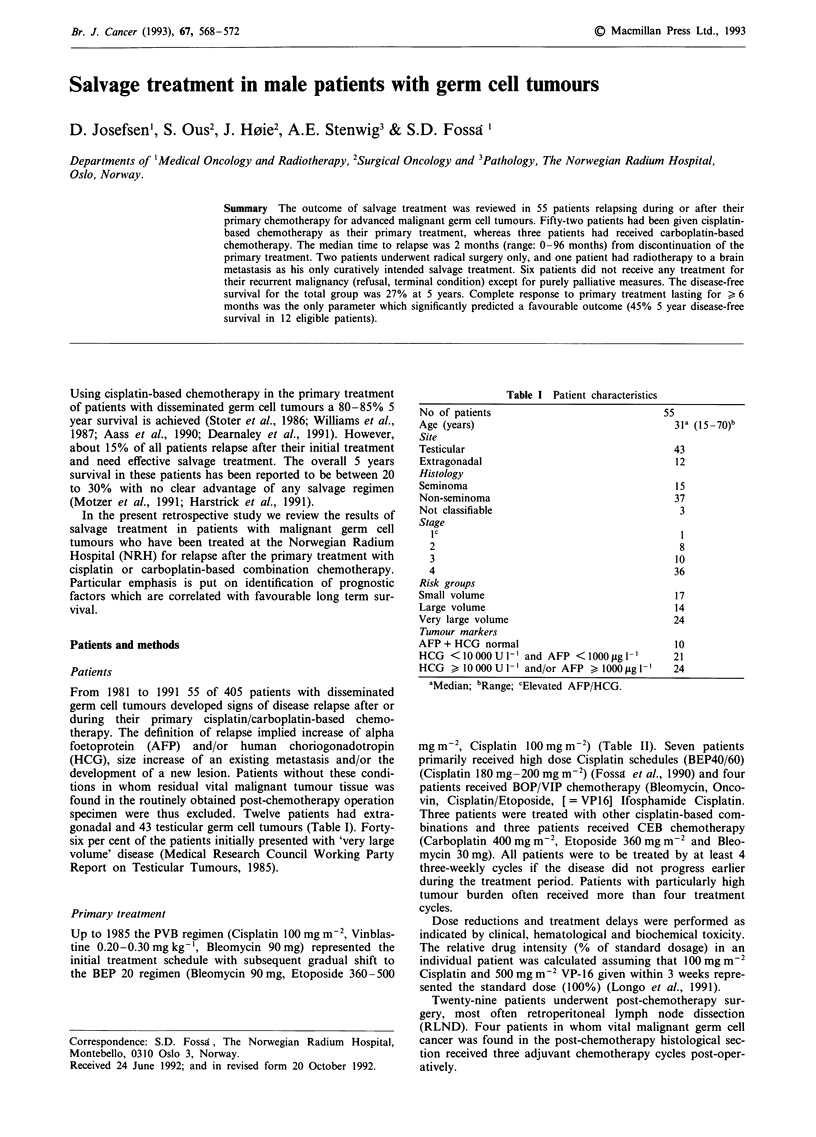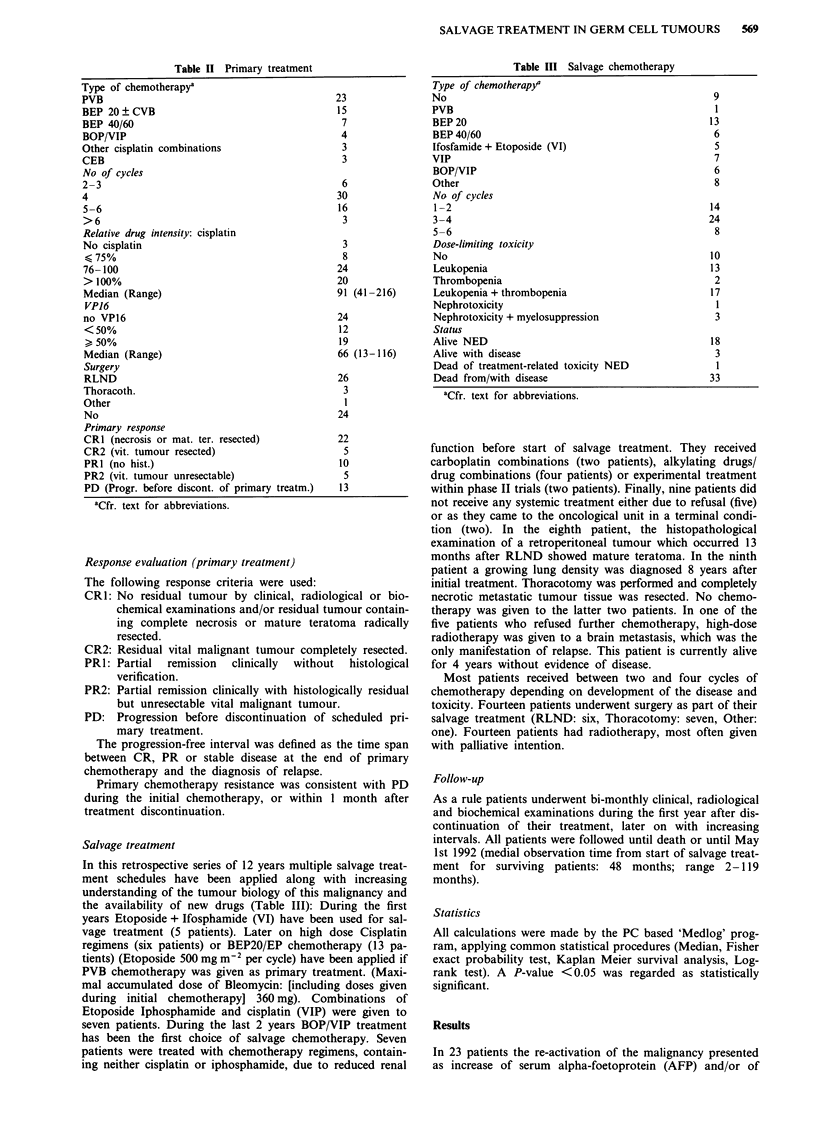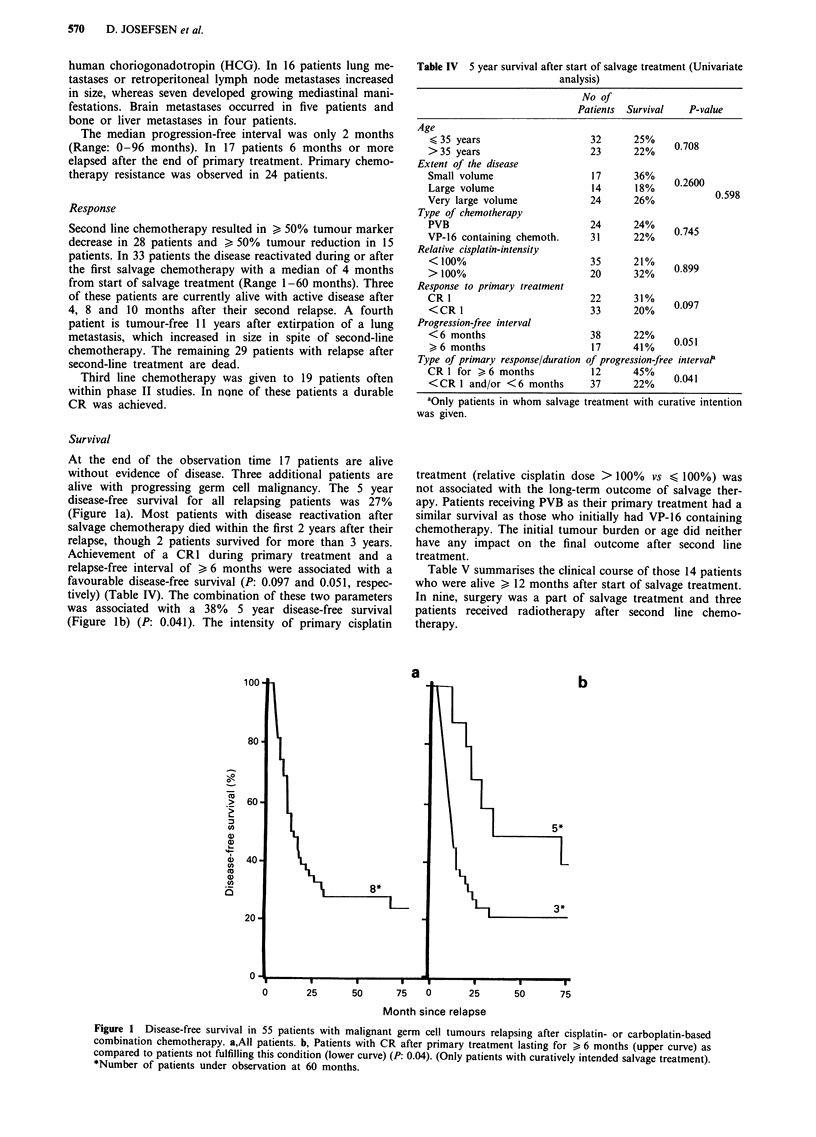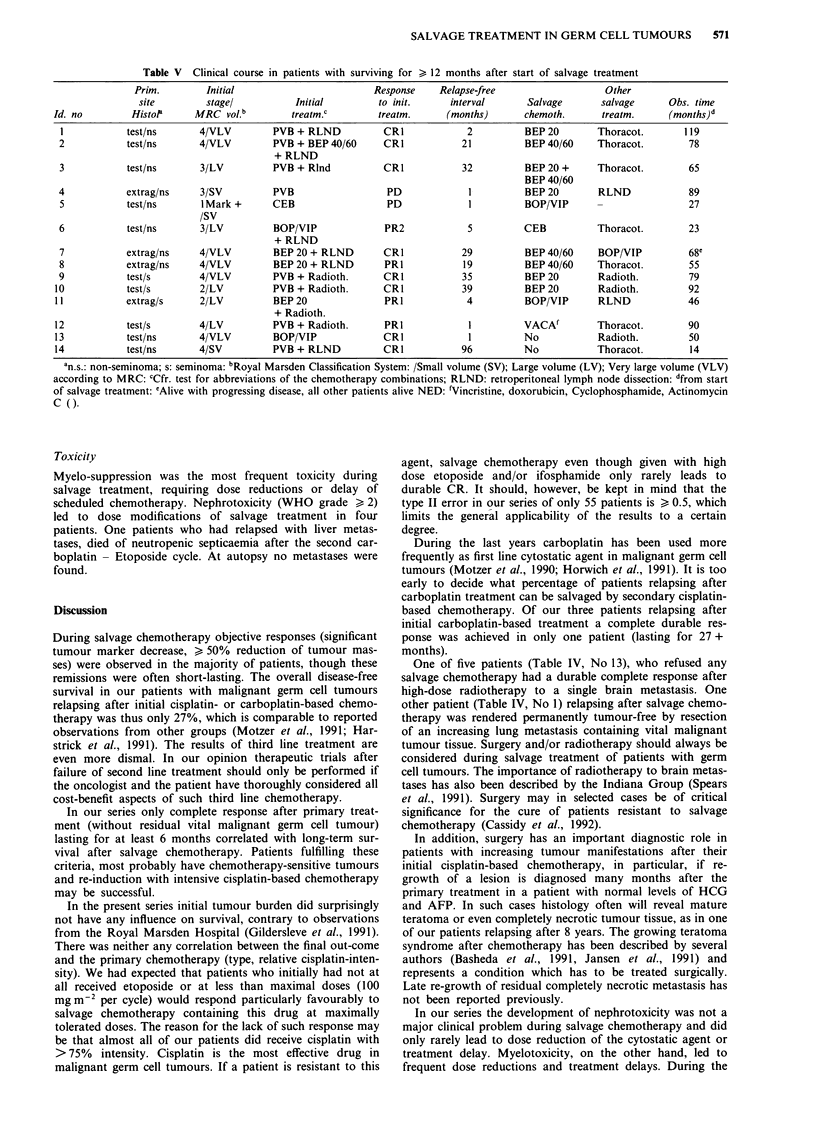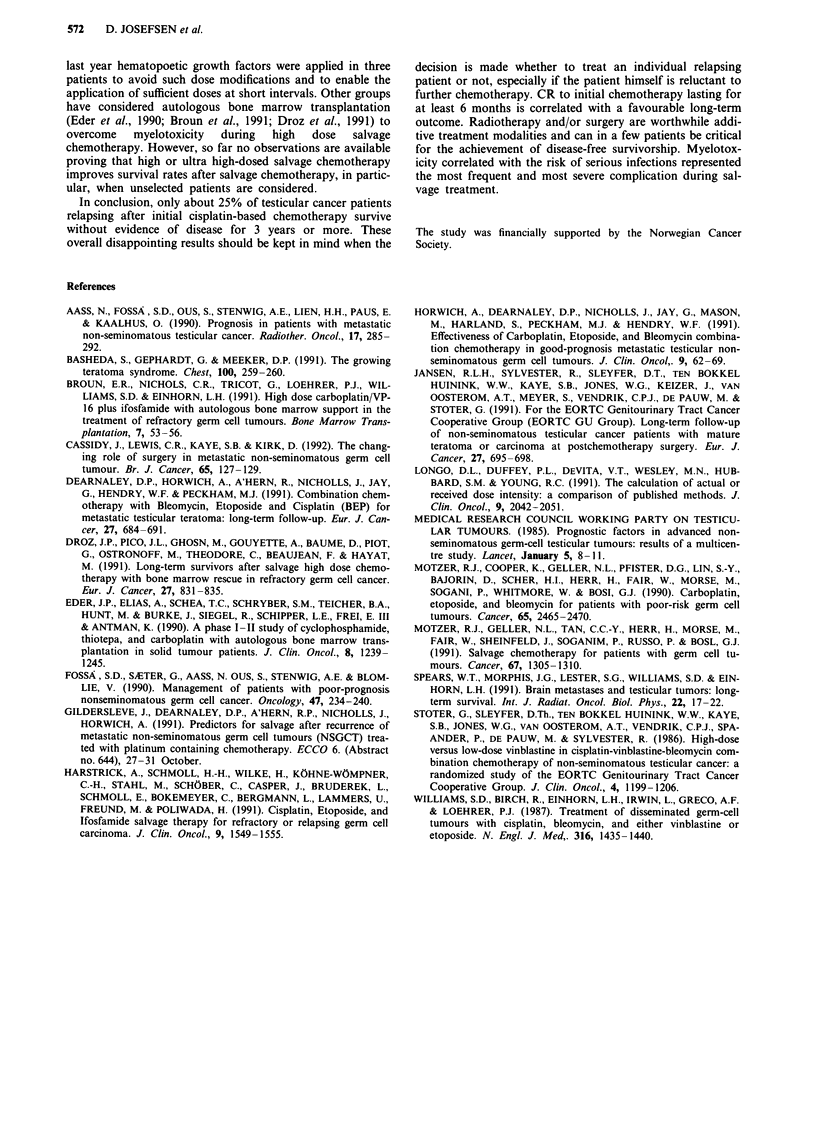# Salvage treatment in male patients with germ cell tumours.

**DOI:** 10.1038/bjc.1993.104

**Published:** 1993-03

**Authors:** D. Josefsen, S. Ous, J. Høie, A. E. Stenwig, S. D. Fosså

**Affiliations:** Departaent of Medical Oncology and Radiotherapy, Norwegian Radium Hospital, Oslo.

## Abstract

The outcome of salvage treatment was reviewed in 55 patients relapsing during or after their primary chemotherapy for advanced malignant germ cell tumours. Fifty-two patients had been given cisplatin-based chemotherapy as their primary treatment, whereas three patients had received carboplatin-based chemotherapy. The median time to relapse was 2 months (range: 0-96 months) from discontinuation of the primary treatment. Two patients underwent radical surgery only, and one patient had radiotherapy to a brain metastasis as his only curatively intended salvage treatment. Six patients did not receive any treatment for their recurrent malignancy (refusal, terminal condition) except for purely palliative measures. The disease-free survival for the total group was 27% at 5 years. Complete response to primary treatment lasting for > or = 6 months was the only parameter which significantly predicted a favourable outcome (45% 5 year disease-free survival in 12 eligible patients).


Br. J. Cancer (1993), 67, 568-572 © Macmillan Press Ltd., 1993

### Salvage treatment in male patients with germ cell tumours

D. Josefsen[1], S. Ous[2], J. Høie[2], A.E. Stenwig[3] & S.D. Fosså[1]

*Departments of [1]Medical Oncology and Radiotherapy, [2]Surgical Oncology and [3]Pathology, The Norwegian Radium Hospital, Oslo, Norway.*

**Summary** The outcome of salvage treatment was reviewed in 55 patients relapsing during or after their primary chemotherapy for advanced malignant germ cell tumours. Fifty-two patients had been given cisplatin-based chemotherapy as their primary treatment, whereas three patients had received carboplatin-based chemotherapy. The median time to relapse was 2 months (range: 0-96 months) from discontinuation of the primary treatment. Two patients underwent radical surgery only, and one patient had radiotherapy to a brain metastasis as his only curatively intended salvage treatment. Six patients did not receive any treatment for their recurrent malignancy (refusal, terminal condition) except for purely palliative measures. The disease-free survival for the total group was 27% at 5 years. Complete response to primary treatment lasting for $\geqslant 6$ months was the only parameter which significantly predicted a favourable outcome (45% 5 year disease-free survival in 12 eligible patients).

Using cisplatin-based chemotherapy in the primary treatment of patients with disseminated germ cell tumours a 80-85% 5 year survival is achieved (Stoter *et al.*, 1986; Williams *et al.*, 1987; Aass *et al.*, 1990; Dearnaley *et al.*, 1991). However, about 15% of all patients relapse after their initial treatment and need effective salvage treatment. The overall 5 years survival in these patients has been reported to be between 20 to 30% with no clear advantage of any salvage regimen (Motzer *et al.*, 1991; Harstrick *et al.*, 1991).

In the present retrospective study we review the results of salvage treatment in patients with malignant germ cell tumours who have been treated at the Norwegian Radium Hospital (NRH) for relapse after the primary treatment with cisplatin or carboplatin-based combination chemotherapy. Particular emphasis is put on identification of prognostic factors which are correlated with favourable long term survival.

**Table I** Patient characteristics

| | |
|---|---|
| No of patients | 55 |
| Age (years) | 31[a] (15-70)[b] |
| *Site* | |
| Testicular | 43 |
| Extragonadal | 12 |
| *Histology* | |
| Seminoma | 15 |
| Non-seminoma | 37 |
| Not classifiable | 3 |
| *Stage* | |
| 1[c] | 1 |
| 2 | 8 |
| 3 | 10 |
| 4 | 36 |
| *Risk groups* | |
| Small volume | 17 |
| Large volume | 14 |
| Very large volume | 24 |
| *Tumour markers* | |
| AFP + HCG normal | 10 |
| HCG $< 10\,000$ U $l^{-1}$ and AFP $< 1000\ \mu g\ l^{-1}$ | 21 |
| HCG $\geqslant 10\,000$ U $l^{-1}$ and/or AFP $\geqslant 1000\ \mu g\ l^{-1}$ | 24 |

[a]Median; [b]Range; [c]Elevated AFP/HCG.

### Patients and methods

### *Patients*

From 1981 to 1991 55 of 405 patients with disseminated germ cell tumours developed signs of disease relapse after or during their primary cisplatin/carboplatin-based chemotherapy. The definition of relapse implied increase of alpha foetoprotein (AFP) and/or human choriogonadotropin (HCG), size increase of an existing metastasis and/or the development of a new lesion. Patients without these conditions in whom residual vital malignant tumour tissue was found in the routinely obtained post-chemotherapy operation specimen were thus excluded. Twelve patients had extragonadal and 43 testicular germ cell tumours (Table I). Forty-six per cent of the patients initially presented with 'very large volume' disease (Medical Research Council Working Party Report on Testicular Tumours, 1985).

### *Primary treatment*

Up to 1985 the PVB regimen (Cisplatin 100 mg $m^{-2}$, Vinblastine 0.20-0.30 mg $kg^{-1}$, Bleomycin 90 mg) represented the initial treatment schedule with subsequent gradual shift to the BEP 20 regimen (Bleomycin 90 mg, Etoposide 360-500 mg $m^{-2}$, Cisplatin 100 mg $m^{-2}$) (Table II). Seven patients primarily received high dose Cisplatin schedules (BEP40/60) (Cisplatin 180 mg-200 mg $m^{-2}$) (Fosså *et al.*, 1990) and four patients received BOP/VIP chemotherapy (Bleomycin, Oncovin, Cisplatin/Etoposide, [ = VP16] Ifosphamide Cisplatin. Three patients were treated with other cisplatin-based combinations and three patients received CEB chemotherapy (Carboplatin 400 mg $m^{-2}$, Etoposide 360 mg $m^{-2}$ and Bleomycin 30 mg). All patients were to be treated by at least 4 three-weekly cycles if the disease did not progress earlier during the treatment period. Patients with particularly high tumour burden often received more than four treatment cycles.

Dose reductions and treatment delays were performed as indicated by clinical, hematological and biochemical toxicity. The relative drug intensity (% of standard dosage) in an individual patient was calculated assuming that 100 mg $m^{-2}$ Cisplatin and 500 mg $m^{-2}$ VP-16 given within 3 weeks represented the standard dose (100%) (Longo *et al.*, 1991).

Twenty-nine patients underwent post-chemotherapy surgery, most often retroperitoneal lymph node dissection (RLND). Four patients in whom vital malignant germ cell cancer was found in the post-chemotherapy histological section received three adjuvant chemotherapy cycles post-operatively.

Correspondence: S.D. Fosså, The Norwegian Radium Hospital, Montebello, 0310 Oslo 3, Norway.
Received 24 June 1992; and in revised form 20 October 1992.

**Table II** Primary treatment

| | |
|---|---|
| Type of chemotherapy[a] | |
| PVB | 23 |
| BEP 20 ± CVB | 15 |
| BEP 40/60 | 7 |
| BOP/VIP | 4 |
| Other cisplatin combinations | 3 |
| CEB | 3 |
| *No of cycles* | |
| 2–3 | 6 |
| 4 | 30 |
| 5–6 | 16 |
| >6 | 3 |
| *Relative drug intensity*: cisplatin | |
| No cisplatin | 3 |
| ⩽ 75% | 8 |
| 76–100 | 24 |
| > 100% | 20 |
| Median (Range) | 91 (41–216) |
| *VP16* | |
| no VP16 | 24 |
| < 50% | 12 |
| ⩾ 50% | 19 |
| Median (Range) | 66 (13–116) |
| *Surgery* | |
| RLND | 26 |
| Thoracoth. | 3 |
| Other | 1 |
| No | 24 |
| *Primary response* | |
| CR1 (necrosis or mat. ter. resected) | 22 |
| CR2 (vit. tumour resected) | 5 |
| PR1 (no hist.) | 10 |
| PR2 (vit. tumour unresectable) | 5 |
| PD (Progr. before discont. of primary treatm.) | 13 |

[a]Cfr. text for abbreviations.

*Response evaluation (primary treatment)*

The following response criteria were used:

CR1: No residual tumour by clinical, radiological or biochemical examinations and/or residual tumour containing complete necrosis or mature teratoma radically resected.

CR2: Residual vital malignant tumour completely resected.

PR1: Partial remission clinically without histological verification.

PR2: Partial remission clinically with histologically residual but unresectable vital malignant tumour.

PD: Progression before discontinuation of scheduled primary treatment.

The progression-free interval was defined as the time span between CR, PR or stable disease at the end of primary chemotherapy and the diagnosis of relapse.

Primary chemotherapy resistance was consistent with PD during the initial chemotherapy, or within 1 month after treatment discontinuation.

*Salvage treatment*

In this retrospective series of 12 years multiple salvage treatment schedules have been applied along with increasing understanding of the tumour biology of this malignancy and the availability of new drugs (Table III): During the first years Etoposide + Ifosphamide (VI) have been used for salvage treatment (5 patients). Later on high dose Cisplatin regimens (six patients) or BEP20/EP chemotherapy (13 patients) (Etoposide 500 mg $m^{-2}$ per cycle) have been applied if PVB chemotherapy was given as primary treatment. (Maximal accumulated dose of Bleomycin: [including doses given during initial chemotherapy] 360 mg). Combinations of Etoposide Iphosphamide and cisplatin (VIP) were given to seven patients. During the last 2 years BOP/VIP treatment has been the first choice of salvage chemotherapy. Seven patients were treated with chemotherapy regimens, containing neither cisplatin or iphosphamide, due to reduced renal

**Table III** Salvage chemotherapy

| | |
|---|---|
| *Type of chemotherapy*[a] | |
| No | 9 |
| PVB | 1 |
| BEP 20 | 13 |
| BEP 40/60 | 6 |
| Ifosfamide + Etoposide (VI) | 5 |
| VIP | 7 |
| BOP/VIP | 6 |
| Other | 8 |
| *No of cycles* | |
| 1–2 | 14 |
| 3–4 | 24 |
| 5–6 | 8 |
| *Dose-limiting toxicity* | |
| No | 10 |
| Leukopenia | 13 |
| Thrombopenia | 2 |
| Leukopenia + thrombopenia | 17 |
| Nephrotoxicity | 1 |
| Nephrotoxicity + myelosuppression | 3 |
| *Status* | |
| Alive NED | 18 |
| Alive with disease | 3 |
| Dead of treatment-related toxicity NED | 1 |
| Dead from/with disease | 33 |

[a]Cfr. text for abbreviations.

function before start of salvage treatment. They received carboplatin combinations (two patients), alkylating drugs/drug combinations (four patients) or experimental treatment within phase II trials (two patients). Finally, nine patients did not receive any systemic treatment either due to refusal (five) or as they came to the oncological unit in a terminal condition (two). In the eighth patient, the histopathological examination of a retroperitoneal tumour which occurred 13 months after RLND showed mature teratoma. In the ninth patient a growing lung density was diagnosed 8 years after initial treatment. Thoracotomy was performed and completely necrotic metastatic tumour tissue was resected. No chemotherapy was given to the latter two patients. In one of the five patients who refused further chemotherapy, high-dose radiotherapy was given to a brain metastasis, which was the only manifestation of relapse. This patient is currently alive for 4 years without evidence of disease.

Most patients received between two and four cycles of chemotherapy depending on development of the disease and toxicity. Fourteen patients underwent surgery as part of their salvage treatment (RLND: six, Thoracotomy: seven, Other: one). Fourteen patients had radiotherapy, most often given with palliative intention.

*Follow-up*

As a rule patients underwent bi-monthly clinical, radiological and biochemical examinations during the first year after discontinuation of their treatment, later on with increasing intervals. All patients were followed until death or until May 1st 1992 (medial observation time from start of salvage treatment for surviving patients: 48 months; range 2–119 months).

*Statistics*

All calculations were made by the PC based 'Medlog' program, applying common statistical procedures (Median, Fisher exact probability test, Kaplan Meier survival analysis, Log-rank test). A $P$-value $<0.05$ was regarded as statistically significant.

### Results

In 23 patients the re-activation of the malignancy presented as increase of serum alpha-foetoprotein (AFP) and/or of human choriogonadotropin (HCG). In 16 patients lung metastases or retroperitoneal lymph node metastases increased in size, whereas seven developed growing mediastinal manifestations. Brain metastases occurred in five patients and bone or liver metastases in four patients.

The median progression-free interval was only 2 months (Range: 0–96 months). In 17 patients 6 months or more elapsed after the end of primary treatment. Primary chemotherapy resistance was observed in 24 patients.

### *Response*

Second line chemotherapy resulted in ≥ 50% tumour marker decrease in 28 patients and ≥ 50% tumour reduction in 15 patients. In 33 patients the disease reactivated during or after the first salvage chemotherapy with a median of 4 months from start of salvage treatment (Range 1–60 months). Three of these patients are currently alive with active disease after 4, 8 and 10 months after their second relapse. A fourth patient is tumour-free 11 years after extirpation of a lung metastasis, which increased in size in spite of second-line chemotherapy. The remaining 29 patients with relapse after second-line treatment are dead.

Third line chemotherapy was given to 19 patients often within phase II studies. In none of these patients a durable CR was achieved.

### *Survival*

At the end of the observation time 17 patients are alive without evidence of disease. Three additional patients are alive with progressing germ cell malignancy. The 5 year disease-free survival for all relapsing patients was 27% (Figure 1a). Most patients with disease reactivation after salvage chemotherapy died within the first 2 years after their relapse, though 2 patients survived for more than 3 years. Achievement of a CR1 during primary treatment and a relapse-free interval of ≥ 6 months were associated with a favourable disease-free survival (*P*: 0.097 and 0.051, respectively) (Table IV). The combination of these two parameters was associated with a 38% 5 year disease-free survival (Figure 1b) (*P*: 0.041). The intensity of primary cisplatin treatment (relative cisplatin dose > 100% *vs* ≤ 100%) was not associated with the long-term outcome of salvage therapy. Patients receiving PVB as their primary treatment had a similar survival as those who initially had VP-16 containing chemotherapy. The initial tumour burden or age did neither have any impact on the final outcome after second line treatment.

Table V summarises the clinical course of those 14 patients who were alive ≥ 12 months after start of salvage treatment. In nine, surgery was a part of salvage treatment and three patients received radiotherapy after second line chemotherapy.

**Table IV** 5 year survival after start of salvage treatment (Univariate analysis)

| | *No of Patients* | *Survival* | *P-value* | |
|---|---|---|---|---|
| *Age* | | | | |
| ≤ 35 years | 32 | 25% | 0.708 | |
| > 35 years | 23 | 22% | | |
| *Extent of the disease* | | | | |
| Small volume | 17 | 36% | 0.2600 | |
| Large volume | 14 | 18% | | 0.598 |
| Very large volume | 24 | 26% | | |
| *Type of chemotherapy* | | | | |
| PVB | 24 | 24% | 0.745 | |
| VP-16 containing chemoth. | 31 | 22% | | |
| *Relative cisplatin-intensity* | | | | |
| < 100% | 35 | 21% | 0.899 | |
| > 100% | 20 | 32% | | |
| *Response to primary treatment* | | | | |
| CR 1 | 22 | 31% | 0.097 | |
| < CR 1 | 33 | 20% | | |
| *Progression-free interval* | | | | |
| < 6 months | 38 | 22% | 0.051 | |
| ≥ 6 months | 17 | 41% | | |
| *Type of primary response/duration of progression-free interval*[a] | | | | |
| CR 1 for ≥ 6 months | 12 | 45% | 0.041 | |
| < CR 1 and/or < 6 months | 37 | 22% | | |

[a]Only patients in whom salvage treatment with curative intention was given.

**Figure 1** Disease-free survival in 55 patients with malignant germ cell tumours relapsing after cisplatin- or carboplatin-based combination chemotherapy. **a**, All patients. **b**, Patients with CR after primary treatment lasting for ≥ 6 months (upper curve) as compared to patients not fulfilling this condition (lower curve) (*P*: 0.04). (Only patients with curatively intended salvage treatment). *Number of patients under observation at 60 months.

**Table V** Clinical course in patients with surviving for ⩾12 months after start of salvage treatment

| *Id. no* | *Prim. site Histol*[a] | *Initial stage/ MRC vol.*[b] | *Initial treatm.*[c] | *Response to init. treatm.* | *Relapse-free interval (months)* | *Salvage chemoth.* | *Other salvage treatm.* | *Obs. time (months)*[d] |
|---|---|---|---|---|---|---|---|---|
| 1 | test/ns | 4/VLV | PVB + RLND | CR1 | 2 | BEP 20 | Thoracot. | 119 |
| 2 | test/ns | 4/VLV | PVB + BEP 40/60 + RLND | CR1 | 21 | BEP 40/60 | Thoracot. | 78 |
| 3 | test/ns | 3/LV | PVB + Rlnd | CR1 | 32 | BEP 20 + BEP 40/60 | Thoracot. | 65 |
| 4 | extrag/ns | 3/SV | PVB | PD | 1 | BEP 20 | RLND | 89 |
| 5 | test/ns | 1Mark + /SV | CEB | PD | 1 | BOP/VIP | – | 27 |
| 6 | test/ns | 3/LV | BOP/VIP + RLND | PR2 | 5 | CEB | Thoracot. | 23 |
| 7 | extrag/ns | 4/VLV | BEP 20 + RLND | CR1 | 29 | BEP 40/60 | BOP/VIP | 68[e] |
| 8 | extrag/ns | 4/VLV | BEP 20 + RLND | PR1 | 19 | BEP 40/60 | Thoracot. | 55 |
| 9 | test/s | 4/VLV | PVB + Radioth. | CR1 | 35 | BEP 20 | Radioth. | 79 |
| 10 | test/s | 2/LV | PVB + Radioth. | CR1 | 39 | BEP 20 | Radioth. | 92 |
| 11 | extrag/s | 2/LV | BEP 20 + Radioth. | PR1 | 4 | BOP/VIP | RLND | 46 |
| 12 | test/s | 4/LV | PVB + Radioth. | PR1 | 1 | VACA[f] | Thoracot. | 90 |
| 13 | test/ns | 4/VLV | BOP/VIP | CR1 | 1 | No | Radioth. | 50 |
| 14 | test/ns | 4/SV | PVB + RLND | CR1 | 96 | No | Thoracot. | 14 |

[a]n.s.: non-seminoma; s: seminoma: [b]Royal Marsden Classification System: /Small volume (SV); Large volume (LV); Very large volume (VLV) according to MRC: [c]Cfr. test for abbreviations of the chemotherapy combinations; RLND: retroperitoneal lymph node dissection: [d]from start of salvage treatment: [e]Alive with progressing disease, all other patients alive NED: [f]Vincristine, doxorubicin, Cyclophosphamide, Actinomycin C ().

### *Toxicity*

Myelo-suppression was the most frequent toxicity during salvage treatment, requiring dose reductions or delay of scheduled chemotherapy. Nephrotoxicity (WHO grade ⩾2) led to dose modifications of salvage treatment in four patients. One patients who had relapsed with liver metastases, died of neutropenic septicaemia after the second carboplatin – Etoposide cycle. At autopsy no metastases were found.

### Discussion

During salvage chemotherapy objective responses (significant tumour marker decrease, ⩾50% reduction of tumour masses) were observed in the majority of patients, though these remissions were often short-lasting. The overall disease-free survival in our patients with malignant germ cell tumours relapsing after initial cisplatin- or carboplatin-based chemotherapy was thus only 27%, which is comparable to reported observations from other groups (Motzer *et al.*, 1991; Harstrick *et al.*, 1991). The results of third line treatment are even more dismal. In our opinion therapeutic trials after failure of second line treatment should only be performed if the oncologist and the patient have thoroughly considered all cost-benefit aspects of such third line chemotherapy.

In our series only complete response after primary treatment (without residual vital malignant germ cell tumour) lasting for at least 6 months correlated with long-term survival after salvage chemotherapy. Patients fulfilling these criteria, most probably have chemotherapy-sensitive tumours and re-induction with intensive cisplatin-based chemotherapy may be successful.

In the present series initial tumour burden did surprisingly not have any influence on survival, contrary to observations from the Royal Marsden Hospital (Gildersleve *et al.*, 1991). There was neither any correlation between the final out-come and the primary chemotherapy (type, relative cisplatin-intensity). We had expected that patients who initially had not at all received etoposide or at less than maximal doses (100 $mg\,m^{-2}$ per cycle) would respond particularly favourably to salvage chemotherapy containing this drug at maximally tolerated doses. The reason for the lack of such response may be that almost all of our patients did receive cisplatin with >75% intensity. Cisplatin is the most effective drug in malignant germ cell tumours. If a patient is resistant to this agent, salvage chemotherapy even though given with high dose etoposide and/or ifosphamide only rarely leads to durable CR. It should, however, be kept in mind that the type II error in our series of only 55 patients is ⩾0.5, which limits the general applicability of the results to a certain degree.

During the last years carboplatin has been used more frequently as first line cytostatic agent in malignant germ cell tumours (Motzer *et al.*, 1990; Horwich *et al.*, 1991). It is too early to decide what percentage of patients relapsing after carboplatin treatment can be salvaged by secondary cisplatin-based chemotherapy. Of our three patients relapsing after initial carboplatin-based treatment a complete durable response was achieved in only one patient (lasting for 27+ months).

One of five patients (Table IV, No 13), who refused any salvage chemotherapy had a durable complete response after high-dose radiotherapy to a single brain metastasis. One other patient (Table IV, No 1) relapsing after salvage chemotherapy was rendered permanently tumour-free by resection of an increasing lung metastasis containing vital malignant tumour tissue. Surgery and/or radiotherapy should always be considered during salvage treatment of patients with germ cell tumours. The importance of radiotherapy to brain metastases has also been described by the Indiana Group (Spears *et al.*, 1991). Surgery may in selected cases be of critical significance for the cure of patients resistant to salvage chemotherapy (Cassidy *et al.*, 1992).

In addition, surgery has an important diagnostic role in patients with increasing tumour manifestations after their initial cisplatin-based chemotherapy, in particular, if re-growth of a lesion is diagnosed many months after the primary treatment in a patient with normal levels of HCG and AFP. In such cases histology often will reveal mature teratoma or even completely necrotic tumour tissue, as in one of our patients relapsing after 8 years. The growing teratoma syndrome after chemotherapy has been described by several authors (Basheda *et al.*, 1991, Jansen *et al.*, 1991) and represents a condition which has to be treated surgically. Late re-growth of residual completely necrotic metastasis has not been reported previously.

In our series the development of nephrotoxicity was not a major clinical problem during salvage chemotherapy and did only rarely lead to dose reduction of the cytostatic agent or treatment delay. Myelotoxicity, on the other hand, led to frequent dose reductions and treatment delays. During the last year hematopoetic growth factors were applied in three patients to avoid such dose modifications and to enable the application of sufficient doses at short intervals. Other groups have considered autologous bone marrow transplantation (Eder *et al.*, 1990; Broun *et al.*, 1991; Droz *et al.*, 1991) to overcome myelotoxicity during high dose salvage chemotherapy. However, so far no observations are available proving that high or ultra high-dosed salvage chemotherapy improves survival rates after salvage chemotherapy, in particular, when unselected patients are considered.

In conclusion, only about 25% of testicular cancer patients relapsing after initial cisplatin-based chemotherapy survive without evidence of disease for 3 years or more. These overall disappointing results should be kept in mind when the decision is made whether to treat an individual relapsing patient or not, especially if the patient himself is reluctant to further chemotherapy. CR to initial chemotherapy lasting for at least 6 months is correlated with a favourable long-term outcome. Radiotherapy and/or surgery are worthwhile additive treatment modalities and can in a few patients be critical for the achievement of disease-free survivorship. Myelotoxicity correlated with the risk of serious infections represented the most frequent and most severe complication during salvage treatment.

The study was financially supported by the Norwegian Cancer Society.

### References

AASS, N., FOSSÅ, S.D., OUS, S., STENWIG, A.E., LIEN, H.H., PAUS, E. & KAALHUS, O. (1990). Prognosis in patients with metastatic non-seminomatous testicular cancer. *Radiother. Oncol.*, **17**, 285–292.

BASHEDA, S., GEPHARDT, G. & MEEKER, D.P. (1991). The growing teratoma syndrome. *Chest*, **100**, 259–260.

BROUN, E.R., NICHOLS, C.R., TRICOT, G., LOEHRER, P.J., WILLIAMS, S.D. & EINHORN, L.H. (1991). High dose carboplatin/VP-16 plus ifosfamide with autologous bone marrow support in the treatment of refractory germ cell tumours. *Bone Marrow Transplantation*, **7**, 53–56.

CASSIDY, J., LEWIS, C.R., KAYE, S.B. & KIRK, D. (1992). The changing role of surgery in metastatic non-seminomatous germ cell tumour. *Br. J. Cancer*, **65**, 127–129.

DEARNALEY, D.P., HORWICH, A., A'HERN, R., NICHOLLS, J., JAY, G., HENDRY, W.F. & PECKHAM, M.J. (1991). Combination chemotherapy with Bleomycin, Etoposide and Cisplatin (BEP) for metastatic testicular teratoma: long-term follow-up. *Eur. J. Cancer*, **27**, 684–691.

DROZ, J.P., PICO, J.L., GHOSN, M., GOUYETTE, A., BAUME, D., PIOT, G., OSTRONOFF, M., THEODORE, C., BEAUJEAN, F. & HAYAT, M. (1991). Long-term survivors after salvage high dose chemotherapy with bone marrow rescue in refractory germ cell cancer. *Eur. J. Cancer*, **27**, 831–835.

EDER, J.P., ELIAS, A., SCHEA, T.C., SCHRYBER, S.M., TEICHER, B.A., HUNT, M. & BURKE, J., SIEGEL, R., SCHIPPER, L.E., FREI, E. III & ANTMAN, K. (1990). A phase I–II study of cyclophosphamide, thiotepa, and carboplatin with autologous bone marrow transplantation in solid tumour patients. *J. Clin. Oncol.*, **8**, 1239–1245.

FOSSÅ, S.D., SÆTER, G., AASS, N. OUS, S., STENWIG, A.E. & BLOMLIE, V. (1990). Management of patients with poor-prognosis nonseminomatous germ cell cancer. *Oncology*, **47**, 234–240.

GILDERSLEVE, J., DEARNALEY, D.P., A'HERN, R.P., NICHOLLS, J., HORWICH, A. (1991). Predictors for salvage after recurrence of metastatic non-seminomatous germ cell tumours (NSGCT) treated with platinum containing chemotherapy. *ECCO* 6. (Abstract no. 644), 27–31 October.

HARSTRICK, A., SCHMOLL, H.-H., WILKE, H., KÖHNE-WÖMPNER, C.-H., STAHL, M., SCHÖBER, C., CASPER, J., BRUDEREK, L., SCHMOLL, E., BOKEMEYER, C., BERGMANN, L., LAMMERS, U., FREUND, M. & POLIWADA, H. (1991). Cisplatin, Etoposide, and Ifosfamide salvage therapy for refractory or relapsing germ cell carcinoma. *J. Clin. Oncol.*, **9**, 1549–1555.

HORWICH, A., DEARNALEY, D.P., NICHOLLS, J., JAY, G., MASON, M., HARLAND, S., PECKHAM, M.J. & HENDRY, W.F. (1991). Effectiveness of Carboplatin, Etoposide, and Bleomycin combination chemotherapy in good-prognosis metastatic testicular non-seminomatous germ cell tumours. *J. Clin. Oncol,*. **9**, 62–69.

JANSEN, R.L.H., SYLVESTER, R., SLEYFER, D.T., TEN BOKKEL HUININK, W.W., KAYE, S.B., JONES, W.G., KEIZER, J., VAN OOSTEROM, A.T., MEYER, S., VENDRIK, C.P.J., DE PAUW, M. & STOTER, G. (1991). For the EORTC Genitourinary Tract Cancer Cooperative Group (EORTC GU Group). Long-term follow-up of non-seminomatous testicular cancer patients with mature teratoma or carcinoma at postchemotherapy surgery. *Eur. J. Cancer*, **27**, 695–698.

LONGO, D.L., DUFFEY, P.L., DEVITA, V.T., WESLEY, M.N., HUBBARD, S.M. & YOUNG, R.C. (1991). The calculation of actual or received dose intensity: a comparison of published methods. *J. Clin. Oncol.*, **9**, 2042–2051.

MEDICAL RESEARCH COUNCIL WORKING PARTY ON TESTICULAR TUMOURS. (1985). Prognostic factors in advanced non-seminomatous germ-cell testicular tumours: results of a multicentre study. *Lancet*, **January 5**, 8–11.

MOTZER, R.J., COOPER, K., GELLER, N.L., PFISTER, D.G., LIN, S.-Y., BAJORIN, D., SCHER, H.I., HERR, H., FAIR, W., MORSE, M., SOGANI, P., WHITMORE, W. & BOSI, G.J. (1990). Carboplatin, etoposide, and bleomycin for patients with poor-risk germ cell tumours. *Cancer*, **65**, 2465–2470.

MOTZER, R.J., GELLER, N.L., TAN, C.C.-Y., HERR, H., MORSE, M., FAIR, W., SHEINFELD, J., SOGANIM, P., RUSSO, P. & BOSL, G.J. (1991). Salvage chemotherapy for patients with germ cell tumours. *Cancer*, **67**, 1305–1310.

SPEARS, W.T., MORPHIS, J.G., LESTER, S.G., WILLIAMS, S.D. & EINHORN, L.H. (1991). Brain metastases and testicular tumors: long-term survival. *Int. J. Radiat. Oncol. Biol. Phys.*, **22**, 17–22.

STOTER, G., SLEYFER, D.Th., TEN BOKKEL HUININK, W.W., KAYE, S.B., JONES, W.G., VAN OOSTEROM, A.T., VENDRIK, C.P.J., SPAANDER, P., DE PAUW, M. & SYLVESTER, R. (1986). High-dose versus low-dose vinblastine in cisplatin-vinblastine-bleomycin combination chemotherapy of non-seminomatous testicular cancer: a randomized study of the EORTC Genitourinary Tract Cancer Cooperative Group. *J. Clin. Oncol.*, **4**, 1199–1206.

WILLIAMS, S.D., BIRCH, R., EINHORN, L.H., IRWIN, L., GRECO, A.F. & LOEHRER, P.J. (1987). Treatment of disseminated germ-cell tumours with cisplatin, bleomycin, and either vinblastine or etoposide. *N. Engl. J. Med,*. **316**, 1435–1440.